# Lithium Induced Parkinsonism: A Case Report

**DOI:** 10.1192/bjo.2022.358

**Published:** 2022-06-20

**Authors:** Tugba Kavasoglu, Ayesha Ahmad, Adnan Khan

**Affiliations:** North Essex Partnership University NHS Foundation Trust, Essex, United Kingdom

## Abstract

**Aims:**

Lithium is an effective mood stabiliser for the treatment of the bipolar disorder. Its utility is not restricted to acute mania and prophylactic treatment of the bipolar disorder. Another well-known indication for its use is the treatment of refractory depression. Lithium can cause several adverse effects, and typically the side effects are dose-related. Unlike antipsychotic medications, lithium is rarely associated with drug-induced Parkinsonism.

**Methods:**

We present a case of 78 years old gentleman who was assessed due to complaints suggestive of cognitive impairment. His past psychiatric history revealed that he was admitted to a psychiatric inpatient unit with a diagnosis of treatment-resistant depression in 1991. Lithium therapy was commenced during this admission, and he remained on lithium for 27 years. The patient was clinically stable in terms of the symptoms of depression; however, he reported bilateral postural tremors 20 years after the initiation of lithium therapy. Initially, he was diagnosed with lithium-induced tremor; however, in the following months, his symptoms had worsened, and he developed new motor disturbances, although the serum levels of lithium were within the therapeutic range. On examination, he had classic parkinsonian signs of shuffling gait, muscle rigidity in all four limbs and freezing of gait. DaT-SPECT imaging clarified the diagnosis as drug-induced Parkinsonism. As the daily lithium dosage was stopped, the patient's motor symptoms improved significantly; nevertheless, some of the symptoms persisted.

**Results:**

The pathophysiological mechanism behind lithium-induced Parkinsonism is unclear. The condition may develop with or without frank lithium toxicity and have diverse presentations. Literature suggests that the risk factors for lithium-induced Parkinsonism appear to be the patients' age, duration of lithium therapy, and serum lithium levels. It has been suggested that older patients have a more permeable blood-brain barrier and decreased renal clearance; hence, serum lithium levels can appear therapeutic, but brain lithium levels may be much higher. Pharmacokinetic drug-drug interactions might also contribute; thus, careful monitoring is essential.

Drug-induced Parkinsonism improves with discontinuation of the offending medication; however, 10% of patients will develop a persistent and progressive parkinsonian syndrome.

**Conclusion:**

This report aims to emphasise the need to consider lithium-induced Parkinsonism when Parkinson Disease symptoms appear in chronic lithium users and close monitoring of lithium levels in geriatric populations. It is essential to recognise the condition, avoid misdiagnosis and prevent inappropriate use of anti-dopaminergic medications.

